# The Housekeeper's Time-Table

**Published:** 1912-06-08

**Authors:** 


### INSTITUTIONAL HOUSEKEEPING.

### The Housekeeper's Time-table.

We give below an outline of the ordinary routine of the housekeeper in a medium-sized hospital, if it is desired effectually to control waste and ensure that the whole establishment be efficiently controlled. The duties enumerated are, it will be seen, of so absorbing and incessant a nature that they must occupy the whole of one woman's time, leaving none over for the even more engrossing functions of the superintendent of nurses and matron of the hospital.

Unless the housekeeper is an early riser she misses one of the most critical hours in the day. It is of the utmost importance that the household should make a good start, and she will do well to be on duty by half-past six at latest, when the domestic staff will already be busily engaged preparing for the early breakfasts. She will personally supervise the arrival of milk cans, meat, vegetables, and other perishable goods, noting their condition, taking samples of milk for testing, and seeing that all is properly stored and weighed. She is there when the pig-tub is emptied and when the refuse is despatched to the incinerator. She visits the porters during their task of filling the coal bunks, and gives directions to inexperienced workers for breaking the coal into proper sized pieces, with due admixture of coke, and for damping the coal-dust for use in banking up fires. Any variation in the amount of coal required the previous day is reported to her, and she receives the key of the coal cellars from the head porter, when he has finished.

After breakfast she visits the store-room and gives out to the cook what she requires for the next twenty-four hours on requisitions she has herself made the previous afternoon. She files a counterfoil for each article, so that when fresh supplies are needed she has a check on the quantities which have been used. After a consultation with the cook she commences her regular inspection of those parts of the hospital and nursing home for which she is responsible, which include every passage, staircase, dining-, sitting-, and bed-room, lavatory and bathroom not included in the patients' quarters, with which latter region she has no concern. She adjusts gas jets and electric burners which may have been left on when the lights were turned out the previous night, and once a week she tests them to see that they are giving a good light, supplying fresh bulbs as required. During her inspection she notes whether the housemaids' work has been properly done, gives instructions to new or careless maids, and notes anything which is out of order, such as a broken window-sash, a loose door-knob, a torn piece of matting, etc. She checks the meter of gas or electric light on each floor, and it is well if she has means to check also the supply of hot water used in the different bathrooms. She checks the time-table of any out-workers who may be employed in any part of the premises, and even the boot cleaning does not escape her observation. She visits the sewing-room, consults with the head sewing-maid, inspects and passes work done the previous day, makes note of any materials required, and sees the book kept for the signatures of the maids who help in this department.

She visits the matron as and when appointed by the latter, reports to her, and receives her instructions.

On special days of the week she prepares orders for articles required for the matron's signature, gives out supplies for the laundry, and issues to each maid the cleaning materials required; gives out household linen, and, if so required by the matron, pays wages and interviews new maids.

After dinner the housekeeper should be off duty till three.

At 3 p.m. the diet-sheets for the next day should have come down from the wards, and the housekeeper visits the kitchen, and with the aid of the cook, after inspection of the larder to see what is left from the dinners, she makes out the requisitions for the different sections of the household for the next twenty-four hours. The menus for the officers' and nurses' tables can be sent up for approval to the matron, who may cross out any item with which she disagrees or insert what she pleases. Orders are then prepared for the tradesmen or contractors, the store-cupboard is visited, new stores are weighed over and their quality carefully tested, note is made of any articles running out of stock, and samples are tested. New servants may have to be instructed in their duties and provided with time-tables, reports written up, and many matters attended to for which in the hurry of the morning there has been no time left. The housekeeper will get some time off duty in the course of the evening, but is responsible for seeing that the house is shut up for the night, and that all lights are out at the appointed hour.

### Housekeeping Queries.

Recipe for Savoury Stew of Vegetable.

Sister W.—*Can you kindly insert a recipe for a vegetable stew, containing haricot beans, very simple yet savoury?*

Will the following suit you? Peel two Spanish onions, cut them into thin rings, and fry them a light yellow in 2 oz. of butter or beef dripping. Shake in 1½ oz. of flour and fry it a light brown. Pour in one quart of water in which 4 oz. of haricot beans have been simmered until nearly tender; the beans should be soaked in the cold water overnight. After adding this vegetable stock, stir the same until boiling, and then add 2 lb. of peeled potatoes, three medium-sized carrots and turnips cut into large cubes, and a cauliflower broken into sprigs. Add the beans and seasoning, and simmer for about one and a half hour, or until the vegetables are tender. Serve neatly on a hot dish with chopped parsley as a garnish. In summer-time substitute green peas and sliced beans for haricots. Tiny spring onions are also a good addition.

To Our Readers.

In this column questions are dealt with relating to cookery and household recipes, the duties of the domestic staff, estimates of quantities required for given numbers, and the cost of diet for patients and staff. Arrangements have been made to deal with samples submitted for examination and report on them.

